# Hypofractionated radiotherapy in head and neck cancer: current evidence and future directions, with a focus on laryngeal cancer

**DOI:** 10.3389/fonc.2026.1953291

**Published:** 2026-09-24

**Authors:** Cristiano Grossi, Orges Spahiu, Giuseppe Riva, Armando Crupi, Paolo G. Arduino, Giancarlo Pecorari, Umberto Ricardi, Giuseppe Carlo Iorio

**Affiliations:** 1 Department of Oncology, School of Medicine, University of Turin, Torino, Italy; 2 Division of Otorhinolaryngology, Department of Surgical Sciences, University of Turin, Turin, Italy; 3 Department of Surgical Sciences, CIR-Dental School, University of Turin, Turin, Italy

**Keywords:** elderly and frail patients, glottic cancer, head and neck cancer, hypofractionated radiotherapy (HFRT), laryngeal cancer, nasopharyngeal cancer, oral cavity cancer, oropharyngeal cancer

## Abstract

Radiotherapy (RT) remains a cornerstone in the management of head and neck squamous cell carcinoma (HNSCC) across both definitive and multimodality treatment settings. While hypofractionated radiotherapy (HFRT) has become an established standard of care for several solid tumors, including prostate, breast, lung, and rectal cancers, its adoption in HNSCC has been more cautious owing to the complex anatomy of the head and neck region, the proximity of numerous critical organs at risk, and concerns regarding severe late toxicity. However, major advances in intensity-modulated radiotherapy (IMRT), volumetric modulated arc therapy (VMAT), image guidance, adaptive radiotherapy, and radiobiological understanding have renewed interest in HFRT as a strategy to maintain oncological efficacy while reducing overall treatment time. Additional drivers include the need to optimize healthcare resource utilization, improve access to radiotherapy in low- and middle-income countries, and minimize hospital visits, as highlighted during the COVID-19 pandemic. Recent prospective evidence has further strengthened the rationale for carefully selected hypofractionated approaches in HNSCC. In elderly and frail patients, the phase III ELAN-RT trial has also provided prospective evidence regarding a tailored hypofractionated approach for patients unable to undergo standard treatment. Among head and neck subsites, laryngeal cancer—particularly early-stage glottic carcinoma—represents the most mature clinical model for HFRT and therefore constitutes the principal focus of this review, while evidence from other HNSCC subsites is examined to assess the broader applicability of hypofractionated treatment strategies. This review critically summarizes the radiobiological rationale, current clinical evidence, technological advances, and ongoing challenges surrounding radical HFRT in HNSCC, with particular emphasis on laryngeal cancer, and discusses future directions for its broader implementation in clinical practice.

## 1 Introduction

Radiotherapy (RT) represents a cornerstone in the management of head and neck squamous cell carcinoma (HNSCC), playing a central role across virtually all disease stages and treatment settings, either alone or in combination with surgery and systemic therapies (1, 2). Over the last decade, hypofractionated radiotherapy (HFRT) has progressively emerged as a standard treatment approach in several solid malignancies. This paradigm shift has been particularly evident in prostate, breast, lung, and rectal cancers, where shorter treatment schedules have demonstrated oncological outcomes and toxicity profiles comparable to those of conventional fractionation, while simultaneously improving patient convenience, treatment accessibility, and healthcare resource utilization (3–11). The primary objective of these strategies has been to preserve treatment efficacy and safety while substantially reducing overall treatment time (12). In HNSCC, however, the implementation of HFRT has historically been more challenging because of the close proximity of target volumes to multiple radiosensitive organs at risk (OARs) and concerns regarding potentially severe late toxicity from larger doses per fraction. Consequently, the delivery of larger doses per fraction raises concerns regarding the risk of severe and potentially irreversible late toxicities, such as soft-tissue necrosis, dysphagia, aspiration, cranial neuropathies, and xerostomia. Furthermore, the radiobiological characteristics of HNSCC and surrounding normal tissues have traditionally favored conventional fractionation schedules, given the relatively low α/β ratio of late-responding tissues and the associated risk of increased late toxicity with dose escalation per fraction.

These concerns are reflected in the substantial burden of treatment-related toxicity historically associated with RT for HNSCC (13, 14). Nevertheless, HFRT remains an area of growing clinical interest, driven by advances in treatment planning, image guidance, intensity-modulated radiotherapy (IMRT), volumetric modulated arc therapy (VMAT), adaptive radiotherapy, and improved understanding of radiobiological principles (12, 13). Such technological developments have enhanced dose conformity and OAR sparing, potentially expanding the therapeutic window for hypofractionated approaches. Interest in HFRT has been further stimulated by global disparities in access to radiotherapy. In low- and middle-income countries (LMICs), where radiotherapy resources are often limited and treatment demand exceeds capacity, shorter treatment schedules may significantly improve access to potentially curative therapy. Moreover, the COVID-19 pandemic highlighted the potential advantages of reducing the number of hospital visits without compromising treatment outcomes. In this context, the recently reported results of the HYPNO (HYPo-fractionated versus NOrmo-fractionated) trial represent an important milestone and provide a strong foundation for further clinical investigation of HFRT in HNSCC (15). Similarly, the randomized phase III ELAN-RT trial provided crucial prospective evidence supporting the feasibility and safety of a dedicated hypofractionated radiotherapy regimen tailored for elderly and frail HNSCC patients who are unsuitable for chemotherapy and conventional standard-fractionation RT (16). Among head and neck malignancies, laryngeal cancer represents a particularly informative model for hypofractionation, with the most mature evidence established in early-stage glottic carcinoma. The main aim of this narrative review is to critically evaluate the current evidence regarding hypofractionated radiotherapy delivered with radical intent across HNSCC, using laryngeal cancer as the principal disease model. Accordingly, the laryngeal literature is examined in greater depth, whereas evidence in oropharyngeal, nasopharyngeal, and oral cavity cancers is discussed to contextualize the extent to which the hypofractionation paradigm may be translated across other HNSCC subsites. Adjuvant radiotherapy, palliative hypofractionation, and re-irradiation strategies (including stereotactic body RT in the re-irradiation setting) are not systematically addressed, although selected evidence is discussed where relevant to the broader interpretation of hypofractionation. Thus, we discuss the radiobiological rationale, available clinical data, ongoing controversies (17, 18), and future directions for hypofractionated treatment strategies.

## 2 Materials and methods

An electronic literature search was conducted in the PubMed database for English articles published up to May 31,2026. Boolean operators (OR, AND) were used to combine the following search terms: “hypofractionation”, “head and neck cancer”, “radiation therapy”, “modern radiation therapy”, “intensity-modulated radiotherapy”, “IMRT”, “HNC”, “volumetric modulated arc therapy”, “vmat”, “late effects”, “radiation induced toxicity”, “toxicity”, “radical radiotherapy”, “SBRT”, “chemoradiotherapy”, “green radiotherapy”. Two independent reviewers (C.G and O.S.) screened titles and abstracts and performed the final article selection. Any discrepancy was resolved by discussion with a third reviewer (G.C.I.). Thereafter, meeting proceedings (European Society of Medical Oncology – ESMO –, European Society for Radiotherapy & Oncology – ESTRO –, American Society of Clinical Oncology – ASCO – and American Society for Radiation Oncology – ASTRO), trial registries (clinicaltrials.gov), reference lists of published studies, review articles and relevant books were also considered. This review was designed as a narrative review supported by a structured literature search rather than as a systematic or scoping review; therefore, a PRISMA-based study selection process was not prospectively performed and corresponding record counts are not available. The bibliographic search was restricted to PubMed, which should be acknowledged as a methodological limitation.

## 3 Results

### 3.1 Radiobiological evidence

Radiobiological modeling and translational research provide important insights into the mechanisms underlying differential responses to fractionation in HNSCC. Using an advanced Monte Carlo simulation model of HNSCC originating from a single cancer stem cell (CSC), Marcu and Marcu demonstrated that hypofractionated radiotherapy may be particularly advantageous in early-stage, well-oxygenated tumors (19). In these tumors, shortening the overall treatment time may mitigate the detrimental effects of accelerated tumor repopulation, a phenomenon largely driven by the CSC compartment. In their model, CSCs represented approximately 5.9% of the pretreatment tumor population and exhibited substantial radioresistance, with a surviving fraction at 2 Gy (SF2) of approximately 77% (19). These assumptions were based on seminal studies identifying CD44^+^ cells as the tumorigenic subpopulation in HNSCC capable of self-renewal and tumor initiation (20), as well as evidence demonstrating enhanced radioresistance among cancer-initiating cells (21). Conversely, the same modeling studies suggested that advanced tumors characterized by severe hypoxia (oxygen tension<6 mmHg) may derive greater benefit from hyperfractionated approaches (19). In this setting, increased opportunities for reoxygenation and cellular redistribution may partially compensate for hypoxia-induced radioresistance, potentially improving tumor control compared with larger fraction sizes (19). However, these findings derive from theoretical radiobiological modeling and remain hypothesis-generating, requiring clinical validation before they can inform fractionation selection. Beyond direct cytotoxic effects, increasing evidence indicates that fractionation schedules may differentially modulate antitumor immunity. *In vitro* studies by Wimmer et al. demonstrated that hypofractionated irradiation (5 × 3 Gy) significantly altered the expression of immune checkpoint molecules in HNSCC cell lines regardless of human papilloma virus (HPV) status (22). Hypofractionation induced upregulation of the immunosuppressive molecules programmed death ligand 1 (PD-L1), PD-L2, and herpesvirus entry mediator (HVEM), whereas the co-stimulatory molecule inducible T-cell costimulator ligand (ICOS-L) was selectively increased in HPV-positive cell lines (22). These findings suggest that radiation dose and fractionation may influence the immunological landscape of HNSCC and potentially affect responsiveness to immune checkpoint blockade. Additional support for this concept comes from the work of Frey et al., who demonstrated that hypofractionated radiotherapy can stimulate dendritic-cell activation and promote coordinated recruitment of innate and adaptive immune populations within the tumor microenvironment (23). Collectively, these preclinical observations suggest that hypofractionated regimens may exert biological effects extending beyond tumor cell kill, influencing both CSC dynamics and immune responses. Such findings provide a rationale for further investigation of the interaction between fractionation and antitumor immunity. However, their clinical relevance in HNSCC and their implications for combination with immunotherapy remain to be established.

### 3.2 Clinical evidence

The following section reviews the clinical evidence on HFRT for laryngeal, oropharyngeal, nasopharyngeal, and oral cavity cancers. The available data are narratively summarized and critically discussed, while the treatment schedules and key findings of the principal studies are presented in **Table 1**.

**Table 1:** Comparative summary of clinical characteristics and key findings of the main HFRT studies.

Study	Hypofractionated schedule	Cancer type	Key findings
Kang et al. 2019 (35) *Prospective*	59.5 Gy to GTV and 47.6 Gy to the remaining larynx/17 fractions or 55 Gy to GTV and 40.7 Gy the remaining larynx/11 fractions	• Glottic tumors cT1-T2	• One dose level not tolerated due to dose-limiting toxicity
Schwartz et al. 2017 (36) *Prospective*	50 Gy/15 fractions or 45 Gy/10 fractions or 42.5 Gy/5 fractions	• Glottic tumors cTis-T2	• SBRT feasible with acceptable acute toxicity
Kovarik et al. 2023 (28) *Prospective*	55 Gy/20 fractions vs 63–65 Gy/30 fractions	• Glottic tumors cT2	• No significant differences in DFS, OS and LRFS • Similar toxicity
Huang et al. 2020 (43) Retrospective	60 Gy/20 fractions	• Oropharynx	• Acceptable locoregional control and disease control rates
Bentzen et al. 2026 (15) *Prospective*	55 Gy/20 fractions vs 66 Gy/33 fractions	• HNSCC (~51% of the cohort was OPSCC)	• Non-inferior to normofractionated RT in terms of locoregional control and late toxicity
Franzese et al. 2020 (48) *Prospective*	69.96-54.45 Gy/33 fractions vs 66–54 Gy/30 fractions	• HNSCC	• No significant late toxicity differences • Higher rate of locoregional progression
Mayo et al. 2022 (46) *Retrospective*	50 or 55 Gy/20 fractions	• HNSCC	• Definitive or postoperative treatment appears safe and well tolerated • No G4 o G5 toxicity
Liu et al. 2023 (50) *Prospective*	60 Gy/25 fractions vs 69.96 Gy/33 fractions	• Nasopharyngeal tumors	• No significant differences in PFS or OS • Similar toxicity

HNSCC, Head and Neck squamous cell carcinoma; OS, overall survival; DFS, disease free survival; PFS- progression free survival; SBRT, stereotactic body radiotherapy; LRFS, local recurrence free survival; HFRT, hypofractionated radiotherapy; GTV, gross tumor volume.

#### 3.2.1 Larynx

Among head and neck malignancies, laryngeal cancer represents the most mature clinical model for the application of hypofractionated radiotherapy. In fact, moderate hypofractionation is currently recognized as a standard treatment option for early-stage glottic cancer and is included among the recommended radiotherapy schedules in current NCCN guidelines (24). The radiobiological rationale for hypofractionation in early glottic carcinoma is supported by evidence demonstrating the importance of both fraction size and overall treatment time (OTT). In a seminal analysis of T1–T2 glottic carcinomas, Le et al. reported that local control in T2 disease was significantly influenced by fractionation parameters, with larger fraction sizes (2.25 Gy) and shorter treatment durations (<43 days) associated with improved 5-year local control rates (25). These findings were subsequently corroborated by Yamazaki et al. in a prospective randomized trial of T1N0M0 glottic carcinoma, which demonstrated superior local control with 2.25 Gy fractions compared with conventional 2 Gy fractions, despite the lower total dose delivered in the hypofractionated arm (26). Additional evidence supporting hypofractionation has emerged from large population-based analyses. Using the National Cancer Database, Bledsoe et al. reported a potential overall survival (OS) advantage associated with hypofractionated schedules in early glottic cancer (27). However, given the retrospective registry-based nature of this analysis, the observed survival difference should be interpreted cautiously because of the potential for selection bias and residual confounding. More recently, Kovarik et al. compared 55 Gy in 20 fractions with conventional regimens delivering 63–65 Gy in 30 fractions for T2N0M0 disease and observed comparable oncological outcomes, with no significant differences in survival or recurrence rates (28). Although not statistically significant, a trend toward reduced acute dysphagia was noted in patients receiving hypofractionated treatment (28). Beyond tumor control, functional preservation remains a major therapeutic objective in laryngeal cancer. Prospective evaluations using the Voice Handicap Index (VHI) have demonstrated favorable long-term voice outcomes following hypofractionated radiotherapy for early glottic cancer (29, 30). These findings are consistent with earlier evidence from altered-fractionation studies showing that shortening OTT may improve local control while also improving voice preservation in patients with laryngeal cancer (31). Collectively, these data support the use of moderate hypofractionation as an effective and function-preserving treatment strategy for early-stage glottic carcinoma. In parallel with changes in dose fractionation, contemporary image-guided techniques have enabled investigation of reduced target volumes for early glottic cancer, particularly single-vocal-cord irradiation (SVCI). Initial experiences established the technical feasibility of limiting irradiation to the involved vocal cord, with encouraging local control and potential reductions in the irradiation of adjacent normal tissues (32). Subsequently, Al-Mamgani et al. evaluated image-guided hypofractionated SVCI (58.08 Gy in 16 fractions) in T1a glottic cancer, reporting excellent local control, limited acute toxicity, and favorable patient-reported voice outcomes (30). These findings were subsequently supported in an expanded cohort of 111 patients, in which 5-year local control and laryngectomy-free survival were 97.1% and 98.1%, respectively, although grade 3 late toxicity occurred in 6.5% of evaluable patients (33). Most recently, a prospective randomized phase II trial compared hypofractionated SVCI (58.08 Gy in 16 fractions) with whole-larynx radiotherapy (63 Gy in 28 fractions) in 57 patients with T1aN0 glottic cancer, reporting comparable local control together with superior voice outcomes and a favorable toxicity profile with SVCI (34). These findings support limited-volume hypofractionated irradiation as a promising strategy for carefully selected T1a glottic cancers, although its implementation requires high-quality image guidance and precise target definition, and further prospective validation remains warranted. Building upon the success of moderate hypofractionation, several groups have explored stereotactic body RT (SBRT) as a means of further reducing treatment duration. Early phase I dose-escalation studies highlighted both the promise and the challenges of this approach. Notably, Kang et al. reported premature closure of one treatment arm because of unexpected dose-limiting toxicities, underscoring the narrow therapeutic window of extreme hypofractionation in the larynx and the exquisite sensitivity of endolaryngeal structures to large fraction sizes (35). Subsequent prospective investigations demonstrated more encouraging results. Schwartz et al. and Sher et al. reported promising local control rates with five-fraction SBRT regimens, accompanied by predominantly grade 1–2 acute toxicities, mainly transient hoarseness and mild dysphagia (36, 37). Importantly, long-term functional assessments suggested preservation of voice quality and a low incidence of severe late toxicity when strict target volume definitions and dosimetric constraints were respected (37). More recently, Sanguineti et al. evaluated an ultra-short regimen of 36 Gy delivered in three fractions for T1 glottic carcinoma in a phase I/II trial (38). The study demonstrated excellent local control, favorable functional outcomes, and no grade 3 or higher acute or late toxicities, further supporting the feasibility of carefully selected ultra-hypofractionated approaches. Nevertheless, given the limited clinical experience and the potentially severe consequences of treatment-related toxicity, SBRT for laryngeal cancer should currently remain restricted to prospective clinical trials led by highly specialized centers with extensive expertise in stereotactic radiotherapy, image guidance, and motion management. Beyond radiobiological and resource-related considerations, shorter treatment duration may also influence the positioning of radiotherapy among available curative options. This is particularly relevant in early glottic cancer, where both radiotherapy and transoral surgical approaches are established treatments. By reducing overall treatment time, moderate hypofractionation may lessen a practical disadvantage of conventionally fractionated radiotherapy and improve convenience for appropriately selected patients. Nevertheless, hypofractionation does not alter the underlying oncological and functional considerations informing the choice between radiotherapy and surgery, and treatment selection should remain based on disease characteristics, expected functional outcomes, and patient preferences.

In contrast to early-stage disease, evidence supporting hypofractionation in locally advanced laryngeal cancer remains limited. Available studies have not demonstrated a clear therapeutic advantage over conventional fractionation. Rawal et al. reported no improvement in tumor response or survival outcomes with hypofractionated chemoradiotherapy compared with conventional schedules (39). Similarly, Roy et al. observed comparable complete response rates but significantly increased acute and late toxicities, including severe mucositis, dermatitis, and swallowing dysfunction, among patients treated with hypofractionated regimens (40). The limitations of treatment intensification were further highlighted by the phase III ART DECO trial. In this study, Nutting et al. evaluated dose-escalated IMRT (67.2 Gy in 28 fractions) versus standard-dose IMRT (65 Gy in 30 fractions) in patients with locally advanced laryngeal and hypopharyngeal cancers and found no improvement in locoregional control with the intensified schedule (41). These findings suggest that simply increasing dose per fraction or biological dose may not translate into improved outcomes in advanced disease, where tumor hypoxia, volume, and treatment-related toxicity remain major therapeutic challenges.

Overall, while moderate hypofractionation is firmly established as a standard treatment option for early-stage glottic cancer, the role of ultra-hypofractionated regimens and hypofractionation in locally advanced laryngeal cancer remains investigational. Future research should focus on patient selection, integration with systemic therapies, and the identification of biological and imaging biomarkers capable of guiding individualized fractionation strategies. Despite the abundance of published evidence, substantial heterogeneity in real-world practice patterns persists, reflecting ongoing uncertainties regarding optimal dose-fractionation schedules across different clinical scenarios (42).

#### 3.2.2 Oropharynx

Oropharyngeal squamous cell carcinoma (OPSCC) represents one of the head and neck subsites in which HFRT has been most extensively investigated. Interest in HFRT has been driven by multiple factors, including the biological rationale for reducing OTT, the need to optimize healthcare resources, and, more recently, the adoption of alternative treatment schedules during the COVID-19 pandemic (43). In addition, the increasing prevalence of HPV-associated OPSCC has stimulated interest in treatment de-escalation strategies aimed at maintaining excellent oncological outcomes while reducing long-term treatment-related morbidity. Regarding the OTT, Fowler estimated that treatment prolongation beyond the planned schedule may result in a loss of local control of approximately 1.4% per day in head and neck cancers (44). Consequently, delivering treatment over 4–5 weeks rather than the conventional 6–7 weeks may partially counterbalance repopulation-related treatment resistance. This concept may be particularly relevant in HPV-negative tumors, which generally exhibit more aggressive biological behavior and lower radiosensitivity compared with HPV-positive disease (45). The COVID-19 pandemic accelerated interest in hypofractionated approaches as a means of reducing patient visits while preserving treatment efficacy. Among the earliest experiences, Huang et al. reported outcomes from Princess Margaret Cancer Centre using a schedule of 60 Gy in 20 fractions for HNSCC, including a substantial proportion of patients with OPSCC (43). The authors observed encouraging locoregional and disease-control outcomes across different T and N categories, supporting the feasibility of this regimen in routine clinical practice (43). Similarly, Mayo et al. demonstrated acceptable acute and late toxicity profiles with HFRT (46), while analysis of the PET-NECK cohort by Vreugdenhil et al. confirmed the feasibility of hypofractionated chemoradiotherapy without unexpected treatment-related toxicities (47). The strongest evidence supporting HFRT in OPSCC and other HNSCC subsites derives from the recently reported phase III HYPNO trial (15). This multicenter study enrolled 792 patients between 2014 and 2020, of whom approximately 51% had OPSCC, and compared a hypofractionated schedule of 55 Gy in 20 fractions with a normofractionated regimen of 66 Gy in 33 fractions. The trial met both primary endpoints, demonstrating non-inferiority for locoregional tumor control (p = 0.04) and grade ≥3 late toxicity (p = 0.004). Three-year locoregional control was 50.7% and 51.2% in the hypofractionated and normofractionated arms, respectively (15). Nevertheless, several features should be considered when extrapolating these findings to contemporary practice. All participating centers were located in low- and middle-income countries, potentially limiting direct applicability to populations with a greater prevalence of favorable-prognosis HPV-associated OPSCC. Moreover, the pragmatic design allowed heterogeneity in radiotherapy planning and delivery techniques, ranging from 2D and 3D conformal RT to IMRT, as well as some institution-dependent aspects of target and prescription definition (15). While these features enhance the relevance of the findings to resource-constrained settings, they also warrant caution when directly extrapolating the results to contemporary IMRT/VMAT-based practice. Successful implementation of HFRT requires careful patient selection and meticulous target volume delineation. Franzese et al., in a cohort comprising more than 50% OPSCC patients, reported higher rates of locoregional progression among patients treated with hypofractionated schedules incorporating wider elective nodal volumes, particularly in low-risk regions (48). These findings emphasize the importance of optimizing target definition and dose distribution when adopting hypofractionated approaches, particularly in anatomically complex regions such as the oropharynx. A major contemporary area of investigation is the use of HFRT within treatment de-escalation strategies for HPV-positive OPSCC. Because these patients generally exhibit excellent survival outcomes and are often younger at diagnosis, reducing long-term treatment-related toxicity has become a key therapeutic objective. As highlighted by Bocha et al., moderate hypofractionation is increasingly being explored as a de-escalation strategy designed to minimize late toxicities—including xerostomia, dysphagia, and swallowing dysfunction—while preserving oncological efficacy (29). Although conventional chemoradiotherapy delivering 70 Gy in 35 fractions remains the standard of care for locally advanced disease, regimens such as 60 Gy in 25 fractions and 55 Gy in 20 fractions are currently being evaluated in prospective studies involving carefully selected low-risk HPV-positive patients (29).

Overall, the available evidence supports continued investigation of HFRT in selected patients with OPSCC. The HYPNO trial has strengthened the broader evidence base for moderate hypofractionation in HNSCC, particularly in resource-constrained settings, although its specific role in oropharyngeal cancer remains to be defined. Ongoing studies will further clarify the potential application of HFRT in OPSCC, particularly within de-escalation strategies for HPV-associated disease, where maintaining oncological efficacy while reducing long-term toxicity remains a major clinical priority.

#### 3.2.3 Nasopharynx

The role of HFRT in nasopharyngeal carcinoma (NPC) remains less clearly defined than in other head and neck cancer subsites. Although advances in IMRT and VMAT have improved dose conformity and organ-at-risk sparing, the application of larger fraction sizes in NPC remains challenging because of the close proximity of critical structures, including the brainstem, optic apparatus, temporal lobes, cochleae, and cranial nerves. Furthermore, the routine use of concurrent cisplatin-based chemotherapy in locally advanced disease raises concerns regarding the potential amplification of acute treatment-related toxicity. Indeed, while shortening the OTT may theoretically enhance tumor control by limiting accelerated repopulation, the combination of hypofractionation and concurrent chemotherapy may increase the risk of acute mucosal toxicity (41). Nevertheless, moderate hypofractionated schedules delivering approximately 2.3–2.5 Gy per fraction have demonstrated encouraging outcomes and acceptable safety profiles in selected clinical settings when delivered with modern IMRT or VMAT techniques and strict adherence to dose constraints for critical OARs. Evidence supporting HFRT in NPC derives primarily from studies in recurrent disease. In a randomized phase II trial, Tian et al. compared hypofractionated IMRT (60 Gy in 27 fractions) with conventional fractionation (68 Gy in 34 fractions) in patients with locally recurrent NPC (49). Although overall survival was numerically higher in the hypofractionated arm, no statistically significant difference was observed. Local failure-free survival was slightly lower following HFRT; however, the incidence of severe mucosal necrosis was significantly reduced, suggesting a potential advantage in terms of late toxicity (49). The potential role of HFRT has also been explored in metastatic NPC. Liu et al. conducted a multicenter prospective randomized phase II trial comparing HFRT plus chemotherapy with conventionally fractionated RT plus chemotherapy in patients presenting with *de novo* metastatic disease (50). Patients received either 60 Gy in 25 fractions or 69.96 Gy in 33 fractions following induction systemic therapy. After a median follow-up of approximately 30 months, no significant differences were observed in progression-free survival, overall survival, locoregional control, or distant metastasis-free survival between the two treatment groups (50). Importantly, both studies were conducted in Chinese centers within an endemic NPC setting, where EBV-associated disease predominates; therefore, the generalizability of these findings to non-endemic populations with different epidemiological and biological characteristics remains uncertain.

Overall, HFRT in NPC should currently be regarded as an investigational approach. While preliminary data suggest that moderate hypofractionation may be feasible in selected patients with recurrent or metastatic disease, robust evidence supporting its routine use in newly diagnosed locally advanced NPC is lacking. Future prospective trials incorporating modern image-guided radiotherapy, adaptive treatment strategies, and biomarker-driven patient selection will be essential to define the optimal role of hypofractionation in this disease.

#### 3.2.4 Oral cavity

The oral cavity remains one of the most challenging head and neck subsites for the implementation of HFRT. This is primarily due to the high radiosensitivity of the oral mucosa, the frequent proximity of target volumes to the mandible, and the consequent risk of severe acute toxicity and osteoradionecrosis (ORN). As a result, surgery followed by conventionally fractionated adjuvant radiotherapy, with or without concurrent chemotherapy, remains the standard of care for most patients with locally advanced oral cavity squamous cell carcinoma (OCSCC). Clinical evidence supporting HFRT in OCSCC is limited. The most relevant prospective data derive from the phase I/II HYPO-ART study, in which Verma et al. evaluated adjuvant HFRT (51). Intermediate-risk patients received 50 Gy over four weeks, whereas high-risk patients were treated with 62.5 Gy over five weeks. No grade 4 toxicities were reported. However, grade 3 mucositis and dysphagia were common, emphasizing the need for intensive supportive care and close multidisciplinary management. Despite these toxicities, the investigators concluded that adjuvant HFRT was feasible and safe when delivered within a structured clinical framework (51). These findings are consistent with earlier retrospective experiences. In the same large Indian cohort discussed above in the laryngeal section, Roy et al. reported favorable locoregional control outcomes with hypofractionated schedules, particularly among patients with oral cavity cancer (40), despite the increased acute and late toxicities observed with hypofractionation in the overall study population. Although these data should be interpreted cautiously given their retrospective nature, they suggest that selected patients with OCSCC may benefit from carefully designed hypofractionated approaches.

Overall, the evidence supporting HFRT in oral cavity cancer remains preliminary. While early prospective and retrospective studies demonstrate feasibility, concerns regarding acute mucosal toxicity and mandibular complications continue to limit widespread adoption. Future investigations should focus on optimizing dose-fractionation schedules, refining patient selection, and integrating modern image-guided radiotherapy techniques to better define the role of HFRT in this setting.

#### 3.2.5 Elderly and frail patients

The potential role of hypofractionated radiotherapy may be particularly relevant in older and frail patients, a population frequently underrepresented in prospective clinical trials and often unable to tolerate prolonged treatment courses. The ELAN-RT trial specifically addressed this population, enrolling patients aged ≥70 years with stage II–IV unresectable HNSCC who were deemed frail based on geriatric assessment (16). Participants were randomized to standard fractionation (70 Gy in 35 fractions over 7 weeks) or a hypofractionated split-course regimen (55 Gy in 20 fractions), delivered in two 2-week courses separated by a planned 2-week treatment-free interval. The hypofractionated regimen met the prespecified non-inferiority criterion for the primary endpoint of being alive without locoregional disease at 6 months and was associated with numerically lower rates of severe acute toxicity. However, overall survival favored standard fractionation, with a median OS of 18.9 months compared with 13.0 months in the hypofractionated arm (16). Importantly, the experimental regimen should be interpreted as split-course hypofractionation rather than continuous HFRT. The planned treatment interruption is radiobiologically relevant in HNSCC, as it occurs during a period in which accelerated tumor repopulation may become increasingly important, potentially counteracting some of the benefits associated with a shorter fractionation schedule. Whether this contributed to the observed difference in overall survival remains unknown and cannot be established from ELAN-RT (16). These findings should therefore not be extrapolated to fit older patients solely on the basis of chronological age. For carefully selected frail patients unable to tolerate standard continuous radiotherapy, the ELAN-RT regimen may represent a pragmatic alternative (16); however, standard fractionation should remain preferred when clinically feasible.

## 4 Conclusion

Hypofractionated radiotherapy represents an increasingly investigated strategy for optimizing the delivery of RT in HNSCC. Advances in treatment planning, image guidance, and radiobiological understanding have renewed interest in abbreviated treatment schedules capable of reducing overall treatment time, improving patient convenience, increasing treatment capacity, and potentially lowering healthcare costs while maintaining oncological efficacy in appropriately selected settings. The strength of evidence supporting HFRT, however, varies considerably across head and neck cancer subsites. Among these, laryngeal cancer—particularly early-stage glottic carcinoma—represents the most mature clinical model, with moderate hypofractionation recognized as a standard treatment approach supported by robust long-term efficacy and functional outcome data. In contrast, evidence in other disease sites remains less definitive. Recent randomized data, including the HYPNO (15) trial, have strengthened the evidence for moderate hypofractionation in selected patients with locally advanced HNSCC, while nasopharyngeal and oral cavity carcinomas remain areas of active investigation with limited evidence. In elderly and frail patients, ELAN-RT (16) supports hypofractionated split-course radiotherapy as a potential alternative when standard treatment is not feasible, although the observed overall survival difference underscores the importance of careful patient selection. Beyond logistical advantages, hypofractionation may have biological implications related to shortening overall treatment time and modulation of antitumor immune responses. However, the clinical relevance of these mechanisms remains incompletely defined, and available radiobiological and preclinical findings should not be directly extrapolated to treatment selection or combinations with immunotherapy. Concerns regarding late toxicity, patient selection, target volume definition, and the optimal integration of systemic therapies further emphasize the need for careful evaluation of HFRT within specific clinical contexts. At present, conventional fractionation remains the standard of care for most patients with HNSCC. Future progress will depend on well-designed prospective randomized trials incorporating subsite-specific analyses, standardized toxicity reporting, patient-reported outcomes, functional endpoints, and long-term follow-up. The identification of biological, molecular, and imaging biomarkers capable of guiding individualized fractionation strategies may further help define which patients are most likely to benefit from hypofractionated approaches. Overall, HFRT represents an established treatment option in selected HNSCC settings, most notably early-stage glottic cancer, while its role across other disease subsites and clinical scenarios remains heterogeneous and, in many cases, investigational. Emerging prospective evidence supports continued evaluation of appropriately selected hypofractionated strategies, but broader implementation should remain guided by disease setting, patient selection, treatment technique, and the maturity of the available clinical evidence.
